# An overload of missense variants in the *OTOG* gene may drive a higher prevalence of familial Meniere disease in the European population

**DOI:** 10.1007/s00439-024-02643-8

**Published:** 2024-03-22

**Authors:** Alberto M. Parra-Perez, Alvaro Gallego-Martinez, Jose A. Lopez-Escamez

**Affiliations:** 1 Division of Otolaryngology, Department of Surgery, Instituto de Investigación Biosanitaria, Ibs.GRANADA, Universidad de Granada, Granada, Spain; 2 Sensorineural Pathology Programme, Centro de Investigación Biomédica en Red en Enfermedades Raras (CIBERER),, Madrid, Spain; 3 Faculty of Medicine and Health, School of Medical Sciences, Meniere’s Disease Neuroscience Research Program, The Kolling Institute, The University of Sydney, 10 Westbourne St, Sydney, NSW Australia

**Keywords:** Meniere’s disease, Hearing loss, Exome sequencing, *OTOG* gene, Population genetics

## Abstract

Meniere disease is a complex inner ear disorder with significant familial aggregation. A differential prevalence of familial MD (FMD) has been reported, being 9–10% in Europeans compared to 6% in East Asians. A broad genetic heterogeneity in FMD has been described, *OTOG* being the most common mutated gene, with a compound heterozygous recessive inheritance. We hypothesize that an *OTOG*-related founder effect may explain the higher prevalence of FMD in the European population. Therefore, the present study aimed to compare the allele frequency (AF) and distribution of *OTOG* rare variants across different populations. For this purpose, the coding regions with high constraint (low density of rare variants) were retrieved in the *OTOG* coding sequence in Non-Finnish European (NFE).. Missense variants (AF < 0.01) were selected from a 100 FMD patient cohort, and their population AF was annotated using gnomAD v2.1. A linkage analysis was performed, and odds ratios were calculated to compare AF between NFE and other populations. Thirteen rare missense variants were observed in 13 FMD patients, with 2 variants (rs61978648 and rs61736002) shared by 5 individuals and another variant (rs117315845) shared by two individuals. The results confirm the observed enrichment of *OTOG* rare missense variants in FMD. Furthermore, eight variants were enriched in the NFE population, and six of them were in constrained regions. Structural modeling predicts five missense variants that could alter the otogelin stability. We conclude that several variants reported in FMD are in constraint regions, and they may have a founder effect and explain the burden of FMD in the European population.

**Supplementary Information:**

The online version contains supplementary material available at 10.1007/s00439-024-02643-8.

## Introduction

Meniere disease (MD [OMIM 156000]) is a chronic inner ear disorder characterized by recurrent episodes of vertigo, low-to-medium-frequency sensorineural hearing loss (SNHL), and tinnitus or aural fullness (Lopez-Escamez et al. 2015). The condition is associated with an accumulation of endolymph in the cochlear duct termed “endolymphatic hydrops” (Semaan et al. 2005), and epidemiological studies have reported an increased prevalence of several comorbidities, such as migraine, respiratory allergic disorders (rhinitis, asthma), and autoimmune/autoinflammatory diseases (Gazquez et al. 2011; Kim et al. 2022a, b; Perez-Carpena and Lopez-Escamez 2019; Radtke et al. 2002).

Meniere disease is more commonly observed in the European population, and familial aggregation has been reported in 9–10% of cases in the European and 6% in the East Asian population (Requena et al. 2014; Lee et al. 2015). There are multiple genes associated with MD (Parra-Perez and Lopez-Escamez 2023), including several SNHL genes, showing autosomal dominant or recessive inheritance (Roman-Naranjo et al. 2020) and digenic inheritance (Roman-Naranjo et al. 2021), supporting genetic heterogeneity in the disease.

The most common gene in familial MD (FMD) is *OTOG* (MIM 604,487), with several Spanish families sharing the same variants with a compound heterozygous recessive inheritance (Roman-Naranjo et al. 2020). Exome sequencing (ES) studies have revealed that a few rare missense variants in the *OTOG* gene may be behind the etiology of a high percentage of FMD cases. Fifteen out of forty-six (33%) Spanish-unrelated MD families with at least one rare missense variant in the *OTOG* gene have been reported. The *OTOG* gene encodes for otogelin, a secreted 2975 amino-acid glycoprotein required for the anchorage of the otolithic and tectorial membranes to the hair cell stereocilia in the sensory epithelia of the vestibule (saccule and utricle) and the organ of Corti. It is also involved in the organization and stabilization of the tectorial membrane structure in the organ of Corti (Avan et al. 2019; Cohen-Salmon et al. 1997).

Previous studies have shown that several non-syndromic SNHL genes have a founder effect in the Asian population (48 variants in 14 genes, 85.7%). However, there are few reported genes showing founder variants in the European population [9 variants in *GJB2* [OMIM 121,011), *TMC1* (OMIM 606,706)*,* and *TMIE* (OMIM 607,237) genes] (Aboagye et al. 2023).

We hypothesize that a founder effect may be related to *OTOG* in FMD, and this burden of rare variants will explain the higher prevalence of FMD in the European population. Therefore, this study aims to compare the frequency and distribution of rare variants in the coding region of *OTOG* across different populations to determine whether there are variants that explain the burden of FMD reported in the European population.

## Methods

### *OTOG* missense rare variants dataset in gnomAD

Exome data from the gnomAD database v.2.1 (Karczewski et al. 2020) were used to retrieve *OTOG* rare missense variants (allelic frequency [AF] < 0.01) for the non-Finnish European (NFE, *N* = 56,885), African/African American (AFR, *N* = 8128), East Asian (EAS, *N* = 9197), South Asian (SAS, *N* = 15,308), Latino/Admixed American (AMR, *N* = 17,296), and global populations (*N* = 125,748).

### *OTOG* missense rare variants dataset in familial MD

We retrieved *OTOG* rare variants (AF gnomAD NFE < 0.01), from ES data previously reported in FMD (Roman-Naranjo et al. 2020). This dataset generated from 100 unrelated FMD patients includes single-nucleotide variants (SNVs) aligned with the reference genome GRCh38/hg38. FMD patients were diagnosed according to the diagnostic criteria established by the International Classification Committee for Vestibular Disorders of the Barany Society (Lopez-Escamez et al. 2015).

To determine whether *OTOG* shows an overload of rare variants in different populations, we selected 64 genes with similar coding sequence (CDS) length to *OTOG* (8778 ± 439 bp) (CCDS76390), which were used as controls (Table S1).

### Variant annotation and ranking

Variants in *OTOG* were assessed using the combined annotation-dependent depletion (CADD) score and SpliceAI score that predicts the impact on splicing processes (Jaganathan et al. 2019). The standards and guidelines described by the American College of Medical Genetics and Genomics (ACMG) and the Association for Molecular Pathology (AMP) were followed (Kim et al. 2022a, b). In addition, selected variants were confirmed in the BAM files to avoid potential false positive calls, using Integrative Genomics Viewer (IGV) (Robinson et al. 2011).

The AF for each variant in the different populations was annotated based on the frequencies described in the gnomAD database v.2.1 (Karczewski et al. 2020). In addition, a GBA was performed using the *OTOG* FMD variants dataset with an AF < 0.01, as previously described (Roman-Naranjo et al. 2020).

### Statistical analysis

The aggregated AF for *OTOG* and control genes was calculated for each population and compared with the AF in the FMD cohort using odds ratios (OR) with a 95% confidence interval (CI). Likewise, frequency comparison was performed using OR between the frequency of variants in the NFE population and all other populations. Variants with a *p *value < 0.05 and OR ≥ 1 were considered enriched in a specific population.

### Linkage analysis

The complete list of *OTOG* common variants (AF > 0.05) was downloaded from the gnomAD database v.2.1 (Karczewski et al. 2020) to calculate the linkage disequilibrium among all known variants in *OTOG,* including variants found in the FMD dataset. The *R*^*2*^ score was obtained and represented for each pair of variants, using the LDmatrix and LDheatmap function from the LDlinkR (Myers et al. 2020) and LDheatmap (Shin et al. 2006) R packages, respectively. Population genotype data used in LDlinkR were obtained from Phase 3 (Version 5) of the 1000 Genomes Project.

### Estimation of missense variant density in the coding sequence

The density of variants in the CDS was calculated to identify regions with an overload of missense variants in the *OTOG* gene. For this, a strategy based on sliding windows was used to calculate the ratio between the window’s variants and length. The chosen window length was 100 bp on each side of the evaluated position, and the step between windows was 1 bp, which resulted in a window of 201 bp length. The missense variants used were those described in the gnomAD v.2.1 database (Karczewski et al. 2020) for each population (NFE, AFR, EAS, SAS, and AMR). The high-density threshold was estimated according to the number of missense variants expected in each population according to gnomAD database v.2.1 (Karczewski et al. 2020), calculating it as a direct proportion of the number of expected variants in the overall population. If the computed density in each window was below the predicted density for a given population, that window was considered a low-density region (LDR) or constrained region.

### Protein modeling

The canonical otogelin amino acid sequence (NP_001264198.1) was retrieved from RefSeq, and the structural model was predicted using AlphaFold2. The quality of the protein structural models was assessed using several structure validation algorithms, such as Molprobity (Williams et al. 2018), ERRAT (Colovos and Yeates 1993), ProSA-web (Wiederstein and Sippl 2007), QMEANDisCo (Studer et al. 2020), and DeepUMQA (Guo et al. 2022). Molprobity calculates an overall score, a weighted logarithmic combination of geometric scores such as clashscore, percentage of unfavored Ramachandran, and bad sidechain rotamers. Lower values indicate better quality. ERRAT analyzes the interactions between atoms and provides an overall quality factor. ProSA-web uses a *z*-score to measure the energy separation between the native fold and the average of an ensemble of the misfolds in standard deviation units of the protein database. In this case, the length of the structure (2925 aa) is outside the experimental length range used in ProSA-web in its protein database; hence, the *z*-score comparison obtained may be limited. QMEANDisCo evaluates the agreement of pairwise distances between residues and distance constraints from homologous structures. DeepUMQA calculates, per residue, the superposition-free score lDDT that assesses the local distance differences of all atoms in a protein structure. The lDDT result is shown as a mean. Higher scores indicate higher quality models.

The *in silico* model was used to predict the protein stability change (ΔΔ*G*) caused by the candidate variants, using the DynaMut2 (Rodrigues et al. 2021), mCSM (Pires et al. 2014), and PremPS (Chen et al. 2020) tools. Variants were classified as neutral when –0.5 < ΔΔ*G*pred < 0.5 (Pancotti et al. 2022). In addition, the pathogenicity prediction of AlphaMissense was calculated for each variant (Cheng et al. 2023).

## Results

### Overload of *OTOG* variants in familiar Meniere disease

Thirteen missense variants with an AF gnomAD NFE < 0.01 were found in 13 Spanish individuals with FMD (Table 1). We confirmed an overload of missense variants in *OTOG* in FMD cases against the NFE population (OR = 3.40 [2.10–5.49], FDR = 5.36E–03) and global population from gnomAD (OR = 3.30 [2.05–5.32], FDR = 9.45E–03).

**Table 1:** Rare missense variants found in the *OTOG* gene for familial Meniere disease (AF < 0.01)

Variant	ID	AA change	CADD	SpliceAI	ACMG	Density region (1)	AF FMD	AF gnomAD NFE	AF gnomAD global
NC_000011.10:g.17553211G > A	rs552304627	NP_001264198.1:p.(Val141Met)	34.0	0.15	Pathogenic (PVS1, PS4, PM2, PP3, BP1)	LDR	0.005 (1/200)	1.34E−03	8.80E−04
NC_000011.10:g.17557227G > A	rs61978648	NP_001264198.1:p.(Val269Ile)	16.59	0	Uncertain significance (PS4, PP1, BP1, BP4)	HDR	0.025 (5/200)	4.43E−03	8.36E−03
NC_000011.10:g.17599671C > T	rs117005078	NP_001264198.1:p.(Pro1240Leu)	33.0	0	Likely pathogenic (PS4, PM2, PP3, BP1)	LDR	0.005 (1/200)	5.55E−03	2.73E−03
NC_000011.10:g.17606001G > A	rs145689709	NP_001264198.1:p.(Arg1353Gln)	23.6	0	Uncertain significance (PS4, PM2, BP1, BP4, BP6)	HDR	0.005 (1/200)	4.08E−03	2.42E−03
NC_000011.10:g.17610645 T > C	rs61744602	NP_001264198.1:p.(Leu1794Pro)	7.32	0	Benign (BS1, BS2, BP1, BP6, BP4)	LDR	0.005 (1/200)	3.59E−03	1.63E−03
NC_000011.10:g.17611118C > T	rs748280789	NP_001264198.1:p.(His1952Tyr)	9.44	0	Uncertain significance (PS4, PM2, BP1, BP3, BP4)	LDR	0.005 (1/200)	3.75E−05	1.36E−05
NC_000011.10:g.17611374C > T	rs61736002	NP_001264198.1:p.(Ala2037Val)	8.51	0.07	Uncertain significance (PS4, PM2, BP1, BP4)	LDR	0.025 (5/200)	1.24E−03	2.47E−03
NC_000011.10:g.17612217G > A	rs188527711	NP_001264198.1:p.(Arg2072His)	27.5	0.04	Likely benign (PM2, BP1, BP4)	LDR	0.005 (1/200)	1.60E−03	7.98E−04
NC_000011.10:g.17635125G > A	rs76461792	NP_001264198.1:p.(Arg2556Gln)	23.0	0.02	Benign (PS4, BS1, BS2, BP1, BP4, BP6)	LDR	0.005 (1/200)	4.89E−03	2.76E−03
NC_000011.10:g.17638480C > A	rs61995750	NP_001264198.1:p.(Gln2621Lys)	14.66	0	Benign (BS1, BS2, BP1, BP4, BP6)	HDR	0.005 (1/200)	2.01E−04	9.84E−04
NC_000011.10:g.17640936C > T	rs567966154	NP_001264198.1:p.(Arg2691Cys)	24.7	0	Benign (BS1, BS2, BP1, BP4, BP6)	LDR	0.005 (1/200)	2.40E−04	8.95−04
NC_000011.10:g.17641065G > A	rs1310923563	NP_001264198.1:p.(Val2734Met)	23.7	0	Uncertain significance (PS4, PM2, BP1, BP4)	HDR	0.005 (1/200)	0	1.39E−05
NC_000011.10:g.17642200G > A	rs117315845	NP_001264198.1:p.(Arg2802His)	16.7	0.03	Uncertain significance (PS4, PM2, BP1, BP4, BP6)	HDR	0.01 (2/200)	2.89E−03	2.61E−03

The notation high-density region (HDR) and low-density region (LDR) indicates the position of the variant in Fig. 4

*AA* amino acid, *ACMG* American College of Medical Genetics and Genomics, *CADD* combined annotation-dependent depletion, *FMD* Familial Meniere disease, *AF* allelic frequency, *NFE* Non-Finnish European

### Comparison of *OTOG* allelic frequencies across different reference populations

**Fig. 1:**
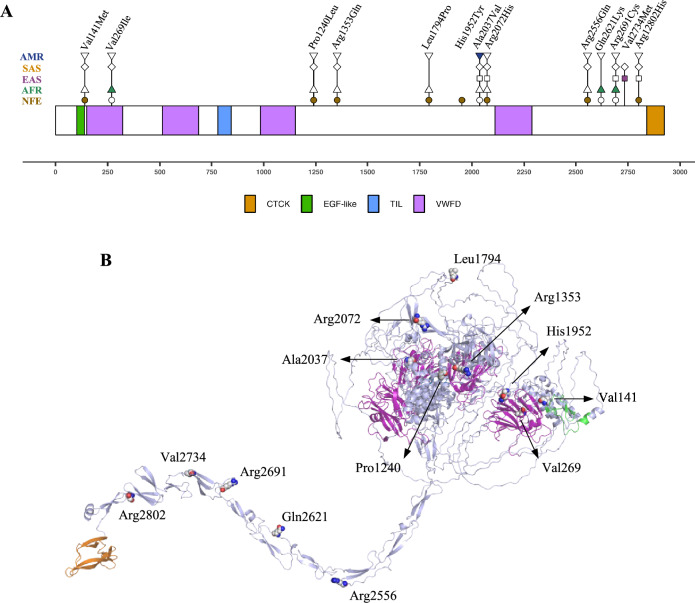
**A** Representation of the otogelin protein sequence showing the rare variants (AF < 0.01) found in the FMD cohort. It is highlighted in which population (NFE, AFR, EAS, SAS, and AMR) they are most frequent. The circle, triangle, square, diamond, and inverted triangle indicate the presence of the variant in the NFE, AFR, EAS, SAS, and AMR populations, respectively. The colored symbol means that that variant is more frequent in that population. **B** Otogelin 3D model outlining the positions of the residues where variants were found. In addition, the different domains that constitute the protein are shown in color. The domains have been colored using the Uniprot domain annotations (Q6ZRI0). CTCK: C-terminal cystine knot-like domain (orange). EGF-like: epidermal growth factor-like domain (green). TIL: trypsin inhibitor-like cysteine-rich domain (blue). VWFD: von Willebrand factor C-like domain (purple). The NP_001264198.1 sequence has been used as a reference to annotate protein positions

The AF of the 13 rare variants observed in the CDS of the FMD cohort was compared between the NFE, AFR, EAS, SAS, and AMR populations (Fig. 1A). Eight rare missense variants were identified to be significantly overrepresented (OR > 1, *p *value < 0.05) in the NFE population compared with the AFR, EAS, SAS, and AMR populations (Table 2). By contrast, five rare missense variants were significantly overrepresented in the AFR (NC_000011.10:g.17557227G > A; NC_000011.10:g.17638480C > A and NC_000011.10:g.17640936C > T), SAS/EAS (NC_000011.10:g.17641065G > A), and AMR (NC_000011.10:g.17611374C > T) populations, when the allelic frequencies were compared with the NFE population (Table 3). Furthermore, it was observed that most variants found in the *OTOG* gene (10/12) were not present in the East Asian population.

**Table 2:** Rare missense FMD *OTOG* variants enriched in the NFE population over the AFR, EAS, SAS, or AMR populations

Variant	AF						AFR			EAS			SAS			AMR		
	NFE	AFR	EAS	SAS	AMR		OR (95% CI)		*P* value	OR (95% CI)		*P *value	OR (95% CI)		*P* value	OR (95% CI)		*p* value
NC_000011.10:g.17553211G > A	1.34E–03	1.48E–04	0	8.88E–05	1.87E–03		9.07	(1.576–362.482)	4.26E–03	–	–	–	15.11	(4.036–127.382)	4.71E-09	0.71	(0.487–1.057)	7.50E-02
NC_000011.10:g.17599671C > T	5.55E–03	8.81E–04	0	7.10E–04	1.42E–03		6.33	(2.873–17.383)	1.64E–09	–	–	–	7.86	(4.759–13.923)	4.54E-28	3.91	(2.752–5.724)	2.05E-19
NC_000011.10:g.17606001G > A	4.08E–03	4.43E–04	0	4.88E–04	2.20E–03		9.24	(3.119–45.202)	2.56E–08	–	–	–	8.39	(4.598–17.053)	2.01E-21	1.86	(1.375–2.552)	2.13E-05
NC_000011.10:g.17610645 T > C	3.59E–03	1.47E–04	0	0	1.02E–03		24.41	(4.332–965.765)	4.08E–09	–	–	–	–	–	-	3.54	(2.326–5.601)	5.88E-12
NC_000011.10:g.17611118C > T	3.75E–05	0	0	0	0		–	–	–	–	–	–	–	–	-	-	-	-
NC_000011.10:g.17612217G > A	1.60E–03	1.47E–04	3.71E–04	2.22E–04	6.92E–04		10.89	(1.903–434.159)	9.20E–04	4.32	(1.628–16.21)	6.14E–04	7.22	(2.981–22.799)	1.46E-08	2.31	(1.366–4.151)	9.40E-04
NC_000011.10:g.17635125G > A	4.89E–03	1.56E–04	0	3.21E–03	1.64E–03		31.53	(5.605–1244.4)	9.72E–12	–	–	–	1.52	(1.167–2.011)	1.48E-03	3.00	(2.136–4.298)	5.91E-13
NC_000011.10:g.17642200G > A	2.89E–03	0	9.28E–05	2.62E–03	2.57E–03		–	–	–	31.26	(5.524–1234.882)	2.03E–11	1.10	(0.812–1.514)	5.50E-01	1.13	(0.835–1.53)	4.66E-01

*AFR* African/African American, *AF* allelic frequency, *AMR* Latino/Admixed American, *CI* confidence interval, *EAS* East Asian, *NF* non-Finnish European, *OR* odds ratios, *SAS* South Asian

**Table 3:** Rare missense FMD *OTOG* variants enriched in the AFR, SAS, EAS, or AMR populations over the NFE population

Variant	AF						AFR			EAS			SAS			AMR		
	NFE	AFR	EAS	SAS	AMR		OR (95% CI)		*P *value	OR (95% CI)		*P* value	OR (95% CI)		*p* value	OR (95% CI)		*p* value
NC_000011.10:g.17557227G > A	4.43E–03	6.28E–02	0	1.42E–03	1.37E–02		0.07	(0.056–0.078)	4.10E–237	–	–	–	3.13	(2.158–4.678)	5.66E–12	0.32	(0.271–0.38)	1.73E–41
NC_000011.10:g.17611374C > T	1.24E–03	4.45E–03	9.34E–05	4.74E–03	4.88E–03		0.28	(0.178–0.442)	1.06E–07	13.25	(2.297–529.908)	1.13E–04	0.26	(0.188–0.357)	2.99E–18	0.25	(0.183–0.343)	3.86E–20
NC_000011.10:g.17638480C > A	2.01E–04	1.56E–02	0	0	9.78E–04		0.01	(0.006–0.024)	4.26E–88	–	–	–	–	–	–	0.21	(0.091–0.435)	7.56E–06
NC_000011.10:g.17640936C > T	2.40E–04	1.27E–02	9.28E–05	8.89E–05	9.37E–04		0.02	(0.01–0.034)	5.87Ev68	2.59	(0.389–109.946)	4.90E–01	2.70	(0.612–24.671)	2.57E–01	0.26	(0.119–0.527)	7.87E–05
NC_000011.10:g.17641065G > A	0	0	9.30E–05	4.44E–05	0		0	–	1	0	(0–7.696)	1.65E–01	0	(0–16.115)	2.92E–01	0	–	1

*AFR* African/African American, *AF* allelic frequency, *AMR* Latino/Admixed American, *CI* confidence interval, *EAS* East Asian, *NFE* Non-Finnish European, *OR* odds ratios, *SAS* South Asian

**Fig. 2:**
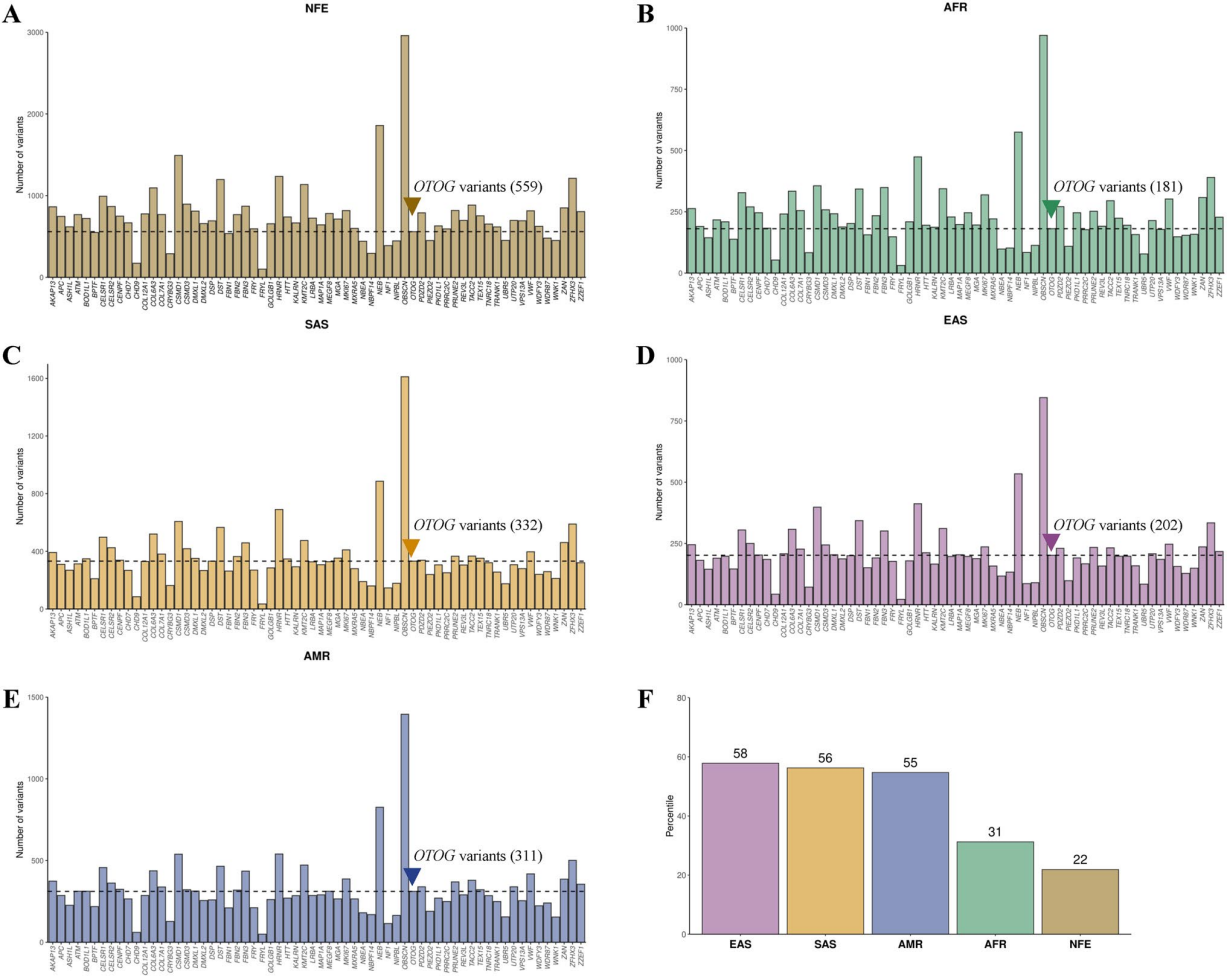
Bar plot showing the number of rare missense variants found in *OTOG* and control genes for each reference population. **A** NFE, **B** AFR, **C** SAS, **D** EAS, **E** AMR, and **F** percentile of the number of rare missense variants in the *OTOG* gene in the NFE, AFR, EAS, SAS, and AMR reference populations compared to the number of rare missense variants in control genes (AF < 0.01)

To determine whether *OTOG* showed an overload of missense rare variants, we selected 64 genes with similar lengths in the CDS to *OTOG*. We ranked the genes according to the number of variants for each reference population (NFE, AFR, EAS, SAS, and AMR), and *OTOG* showed a different burden of rare missense variants in the CDS compared to genes with the same CDS in the different populations (Fig. 2). In the NFE population, the number of variants described was in the 22nd percentile, while for the EAS, SAS, and AMR populations, it was above the 55th percentile.

### Linkage analysis

**Fig. 3:**
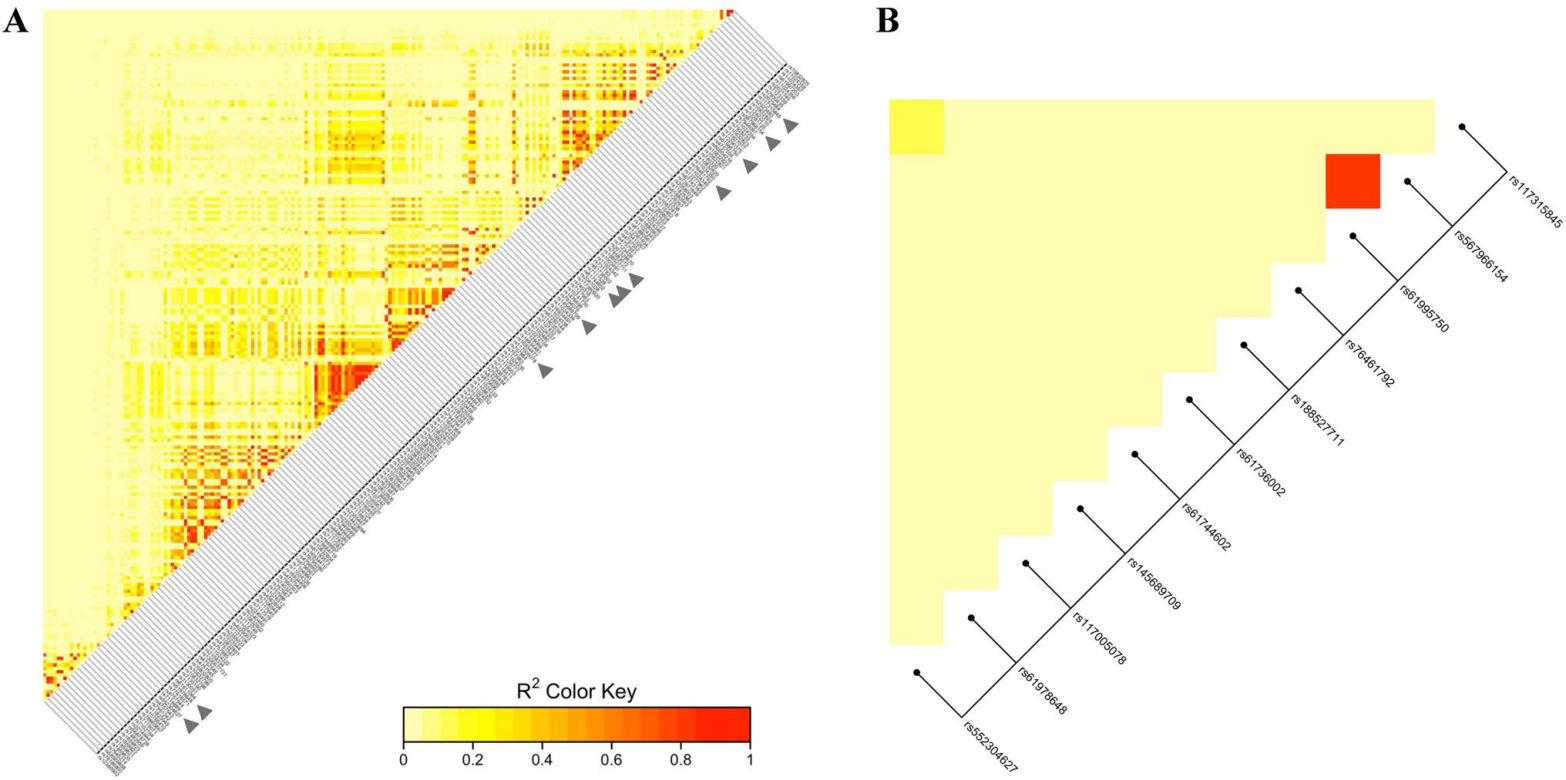
**A** Heatmap showing the pairwise linkage disequilibrium of variants in the *OTOG* gene (MAF > 0.05 plus *OTOG* variants in the FMD cohort) across all populations. Variants with triangular label are the variants found in the FMD cohort. **B** Heatmap representing the pairwise linkage disequilibrium of FMD variants in the *OTOG* gene. The two variants not shown in the plots are not annotated in Phase 3 (Version 5) of the 1000 Genomes Project

The linkage analysis showed that the *OTOG* sequence has a low linkage disequilibrium in the CDS (Fig. 3). Only two variants [NC_000011.10:g.17642200G > A (rs117315845) and NC_000011.10:g.17553211G > A (rs552304627)] were in moderate linkage disequilibrium in the NFE population, with an *R*^2^ of 0.332. These variants have yet to be described in the SAS and EAS populations. Both variants were found in the same MD family.

Two other rare variants were linked in the AFR and AMR populations [NC_000011.10:g.17640936C > T (rs567966154) and NC_000011.10:g.17638480C > A (rs61995750)], with an *R*^2^ of 0.832 and 1, respectively, but not in the NFE, SAS, and EAS populations, since they were not described in the 1000G data. These two variants were found in the same family.

### Variant density analysis in the *OTOG* coding sequence

**Fig. 4:**
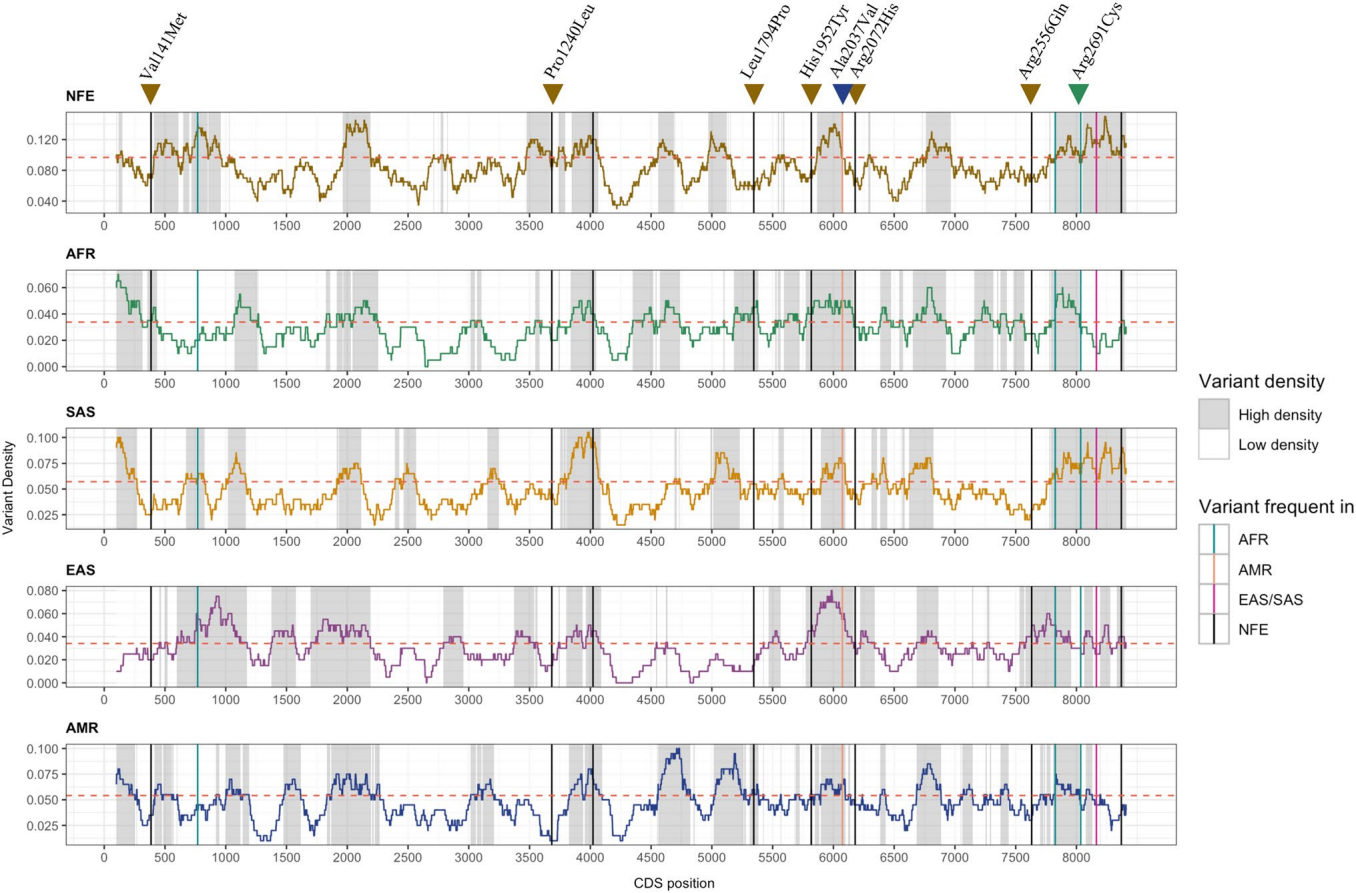
Variant density profile along the OTOG CDS in the NFE, AFR, SAS, EAS, and AMR populations calculated with a 201 bp sliding window. High-density regions (HDR; gray areas) and low-density regions (LDR) are those with a high or low number of variants affecting the value of missense variants expected in each population analyzed according to gnomAD v2.1. The eight variants found in the FMD cohort in low-density regions in the NFE population are indicated with triangles. The triangles’ colors (brown, blue, and green) indicate more frequent variants in NFE, AMR, and AFR, respectively. The Red dashed line indicates the threshold density used for each population according to the number of variants expected in the CDS. Variants in the FMD cohort are represented with vertical lines in each plot. Black lines represent frequent variants in the NFE population, green lines in AFR, pink lines in EAS/SAS, and orange in AMR. The NP_001264198.1 sequence has been used as a reference to annotate protein positions

The distribution of LDR in the *OTOG* CDS was similar when compared across different populations (NFE, AFR, SAS, EAS, and AMR) (Fig. 4). The similarity percentage between the regions of the NFE population compared to SAS, AMR, EAS, and AFR was 73.78%, 66.37%, 65.35%, and 60.18%, respectively.

Five variants (NC_000011.10:g.17557227G > A, NC_000011.10:g.17606001G > A, NC_000011.10:g.17638480C > A, NC_000011.10:g.17641065G > A, and NC_000011.10:g.17642200G > A) were found in high-density regions in the *OTOG* CDS. On the other hand, eight rare variants were identified in constrained regions, including NC_000011.10:g.17611374C > T, shared among four FMD patients.

### Protein modeling

The otogelin protein model used to evaluate the impact of SNVs on protein stability was obtained by AlphaFold2 modeling (Fig. 1B). According to the geometric validation results (Table S2), a trustworthy model has been obtained compared to experimentally solved structures at the geometric level.

The otogelin model is predicted to have a globular structure and a tail, with secondary structure mainly in β-sheets, which ends with a C-terminal cystine knot (CTCK) domain. In the globular region, there are four Von Willebrand factor type D (VWFD) domains, a trypsin inhibitor-like (TIL) domain, and an epidermal growth factor-like (EGF-like) domain, surrounded by several disordered regions (Fig. 1).

The variants NP_001264198.1:p.(Val141Met), NP_001264198.1:p.(His1952Tyr), NP_001264198.1:p.(Ala2037Val), NP_001264198.1:p.(Arg2072His), and NP_001264198.1:p.(Arg2802His) have been predicted in silico to change the otogelin global stability by at least two different methods (Table S3). In this context, the NP_001264198.1:p.(Val141Met), NP_001264198.1:p.(Ala2037Val), NP_001264198.1:p.(Arg2072His), and NP_001264198.1:p.(Arg2802His) variants have been classified as destabilizing. In contrast, the NP_001264198.1:p.(His1952Tyr) variant is classified as stabilizing (Figure S1).

Nevertheless, the remaining eight variants found in FMD patients are classified as neutral according to the predicted perturbation of protein stability.

## Discussion

Our study compares the AF and distribution of *OTOG* rare variants across different populations to assess if the reported FMD variants may have an *OTOG*-related founder effect that would explain the higher prevalence of FMD in NFE population.

MD has a significant familial aggregation in the NFE population, and most reported families with MD have a European ancestry (Requena et al. 2014). Epidemiological studies estimate that the prevalence of FMD is 5–23.5% (Hietikko et al. 2013a, b). In the European population, an overall percentage of 9–15% has been calculated, 9–13% in Spain (Frejo et al. 2017; Requena et al. 2014), 12% in Sweden (Birgerson et al. 1987), and 9.3% in Finland (32.7% for relatives with Meniere-like symptoms) (Hietikko et al. 2013a, b), while in the Asian population, the estimated proportion is approximately 6% in Japan (5.8%) (Mizukoshi et al. 1979) and South Korea (6.3% for relatives with definite FMD and 9.8% FMD-like syndrome) (Lee et al. 2015). The familial aggregation data are likely to be overestimated due to the use of questionnaires and patient interviews and the difficulty of collecting a large cohort of FMD (Morrison et al. 2009), combined with the challenge of MD diagnosis and the different diagnostic criteria in studies published before 1995.

The reported differences in the FMD prevalence may be due to differences in the genetic structure of each population. More frequent and founder variants in hearing impairment genes have already been described in European, African, Asian, and American populations (Aboagye et al. 2023; Adadey et al. 2022); therefore, finding founder variants in specific genes that cause FMD would not be surprising. Founder variants are those variants found with a high frequency within a particular population caused by the presence of the variant in an ancestor or small group of ancestors (Jain et al. 2021).

Among the known FMD genes, *OTOG* seems one of the most relevant, with several Spanish families sharing the same variants with a compound heterozygous recessive inheritance pattern (Roman-Naranjo et al. 2020). *OTOG* is expressed in both vestibular and auditory sensory organs, and Otog knockout mice exhibit both auditory and vestibular phenotypes (Avan et al. 2019), supporting the hypothesis that variants in *OTOG* may contribute to MD development. Furthermore, the relative expression of *OTOG* is higher in the apex than at the base of the cochlea, which may be significant given that hearing loss in MD patients is initially observed at low and medium frequencies (El-Amraoui et al. 2001). For this reason, the *OTOG* gene was chosen as the best candidate to study the allelic frequency of its variants across different populations to test whether *OTOG* could explain the higher prevalence of FMD in the European population and whether there are variants with a founder effect.

Our study shows 13 missense variants in FMD and 8 of them are located in constrained regions in NFE: NC_000011.10:g.17553211G > A (rs552304627), NC_000011.10:g.17599671C > T (rs117005078), NC_000011.10:g.17610645 T > C (rs61744602), NC_000011.10:g.17611118C > T (rs748280789), NC_000011.10:g.17611374C > T (rs61736002), NC_000011.10:g.17612217G > A (rs188527711), NC_000011.10:g.17635125G > A (rs76461792), and NC_000011.10:g.17640936C > T (rs567966154) (Table 1).

Among all variants found, three rare variants are shared in different unrelated FMD patients. The variants NC_000011.10:g.17557227G > A and NC_000011.10:g.17611374C > T are in the same four FMD patients (three heterozygous and one homozygous). Of note, these two variants are also overrepresented in the AFR and AMR populations, and this could be explained by the mixed ancestry of North African and American with the Spanish population. These variants, which are within the same haplotype and have been previously described in FMD patients, could impact the splicing processing and protein stability, increasing the susceptibility to develop a MD-like phenotype. The variant NC_000011.10:g.17557227G > A, although classified as VUS (variant of uncertain significance) by the ACMG criteria, is in the last position of exon 4. This could disrupt the consensus splicing site, generating possible non-functional alternative forms of the protein. Further functional studies would be required since SpliceAI did not predict a splicing defect. In addition, the variant NC_000011.10:g.17611374C > T has been associated in ClinVar (Landrum et al. 2018) with autosomal recessive non-syndromic hearing loss 18B (DFNB18B), supporting its possible pathogenic effect in FMD. None of the variants found in *OTOG* have been described in published families with DFNB18B (Ganaha et al. 2019).

On the other hand, the NC_000011.10:g.17642200G > A variant has been found in two FMD patients. This variant, classified as VUS and enriched in NFE population, is in the otogelin’s tail, close to the CTCK domain, which is involved in the formation of antiparallel homodimers (Avan et al. 2019). Since this variant is predicted to be destabilizing, it could potentially interfere with the correct dimer formation and destabilize the connections between the tectorial and otolithic membranes and the hair bundles of hair cells, as observed in mice (Avan et al. 2019), increasing the susceptibility to MD. However, the CTCK-mediated dimer forms a highly resistant structure, with 11 pairs of cysteine residues forming disulphide bonds (Zhou and Springer 2014), so the effect of the NC_000011.10:g.17642200G > A variant on otogelin functionality is unclear.

The variants NC_000011.10:g.17638480C > A and NC_000011.10:g.17640936C > T, classified as benign and more frequent in the African population, were both found in the same FMD patient and in the same haplotype. Although these variants were found in the otogelin’s tail, they were predicted to have no effect on the protein’s stability, so it was not possible to determine whether they will have a functional impact.

The rest of the variants described were each found in different single FMD patients. Since only the exonic variants have been explored, it cannot be excluded that these patients have a second variant in intronic or expression-regulating regions. Further, given the possibility that MD is a multi-factorial, polygenic disease in which epigenetics also play a role (Flook et al. 2021), we cannot rule out that other variants and genes may increase the risk of MD, as described in Hui et al. (2023) for hearing loss.

According to the population frequency of the 13 variants, 8 have a higher frequency in the NFE population than in the rest of the populations, which may indicate a founder effect of these variants in the European population. Three arguments supports this hypothesis: (a) the pathogenicity CADD score > 20 (Niroula and Vihinen 2019), (b) the predicted change in protein stability, and (c) their occurrence in constrained regions or regions of low variant density, since constrained regions are enriched in pathogenic variants in ClinVar (Havrilla et al. 2019).

The finding of 8/13 enriched variants in the NFE population, could be because the FMD cohort mainly includes Spanish patients. For this reason, the high proportion of Spanish individuals in this cohort may explain the finding of three variants that were more frequent in the African than in the NFE population, since North African ancestry in the Spanish population may be up to 11% (Bycroft et al. 2019). Accordingly, it is possible that the prevalence of FMD could be similar in the North African and Spanish populations; thus, current epidemiological studies of MD and familial aggregation (FMD) are needed to support this hypothesis.

We also found that most of the variants found in *OTOG* in our FMD cohort are not found or have a very low frequency in the East Asian population (EAS). This may be due to the greater genetic divergence between both populations (Wang et al. 2018), but it could also anticipate a lower prevalence of OTOG-mediated FMD in East Asian population.

Some of these missense variants are in the protein’s tail and may interfere with the interaction with other structural proteins or the dimers’ formation; however, the physical interactions of otogelin with other tectorial or otolithic membrane proteins are not known.

## Limitations

This study is based on exome sequencing datasets that found a burden of missense variants in the CDS in the *OTOG* gene in FMD (Parra-Perez and Lopez-Escamez 2023); however, most families remain undiagnosed and the discovery of variants in intronic or intergenic regulatory regions could change the current picture of the genetic architecture of FMD. Therefore, whole genome sequencing studies are needed. Besides, the lack of genetic studies of FMD in other populations (Escalera-Balsera et al. 2020) prevents estimating the genetic contribution of *OTOG* to FMD.

## Conclusions

The *OTOG* gene has an overload of rare missense variants in the NFE population. Several *OTOG* variants observed in FMD are found in constrained regions and could have a founder effect in the NFE population. Further genomic studies on FMD in other populations are needed to know which genes may contribute to its development.

### Supplementary Information

Below is the link to the electronic supplementary material.

Supplementary file1 (PDF 1247 KB)

## Data Availability

The datasets used and/or analyzed during the current study are available from the corresponding author on reasonable request.
