# Supplementary material for: An overload of missense variants in the OTOG gene may drive a higher prevalence of familial Meniere disease in the European population
Source: Hum Genet. 2024 Mar 22;143(3):423–35. doi: 10.1007/s00439-024-02643-8 (PMC11043142; doi:10.1007/s00439-024-02643-8)
Supplement: Supplementary file 1 — Supplementary file1 (PDF 1247 KB) [file 439_2024_2643_MOESM1_ESM.pdf]

Supplementary materials

An overload of missense variants in the *OTOG* gene may drive a higher prevalence of familial Meniere disease in the Spanish population

Alberto M. Parra-Perez[1-3], Alvaro Gallego-Martinez[1-3], Jose A. Lopez-Escamez [1-4]

[1] *Otology & Neurotology Group CTS495, Department of Genomic Medicine, GENYO-Centre for Genomics and Oncological Research–Pfizer/University of Granada/ Junta de Andalucía, PTS, Granada, Spain.*

[2] *Division of Otolaryngology, Department of Surgery, Instituto de Investigación Biosanitaria, ibs.GRANADA, Granada, Universidad de Granada, Granada, Spain*

[3] *Sensorineural Pathology Programme, Centro de Investigación Biomédica en Red en Enfermedades Raras, CIBERER, Madrid, Spain*

[4] *Meniere's Disease Neuroscience Research Program, Faculty of Medicine & Health, School of Medical Sciences, The Kolling Institute, The University of Sydney, Sydney, New South Wales, Australia*

Corresponding Author:

Jose A. Lopez-Escamez

Meniere Disease Neuroscience Lab,

Faculty of Medicine and Health, School of Medical Sciences, The University of Sydney

Rm 611024, Level 11 Kolling Institute | 10 Westbourne St, St Leonards NSW 2065

Phone: +61 423 066 010

Email: jose.lopezescamez@sydney.edu.au

Index **Page**

Table S1 – Control genes with a CDS length similar to *OTOG* CDS (8778 ± 439 bp). 3

Table S2 – Model quality validation of otogelin predicted 3D protein structural model. 4

Table S3 – Protein stability change and Alphamissense prediction prediction caused by missense variants in the otogelin model. 5

Figure S1 – Predicted effect of variants on the otogelin structural model. The 5 variants predicted a change in protein stability ($\Delta\Delta G$), according to mCSM-stability, DynaMut2 and PremPS tools. 6

Table S1. Control genes with a CDS length similar to *OTOG* CDS (8778 ± 439 bp).

Gene	CDS length	Gene	CDS length
ADGRG4	8628	*LRBA*	8592
AKAP13	8454	*MAP1A*	8412
APC	8586	*MEGF8*	8538
ASH1L	8910	*MGA*	9198
ATM	9171	*MKI67*	8691
BOD1L1	9156	*MXRA5*	8487
BPTF	9141	*NBEA*	8904
CELSR1	9060	*NBPF14*	8967
CELSR2	8772	*NEB*	9024
CENPF	9168	*NF1*	8550
CHD7	8994	*NIPBL*	8415
CHD9	8694	*OBSCN*	9033
COL12A1	9192	*PDZD2*	8520
COL6A3	8916	*PIEZO2*	8598
COL7A1	8835	*PKD1L1*	8586
CRYBG3	8913	*PRRC2C*	8697
CSMD1	8902	*PRUNE2*	9189
CSMD3	8934	*REV3L*	9159
DMXL1	9147	*TACC2*	8847
DMXL2	9177	*TEX15*	8370
DSP	8616	*TNRC18*	8907
DST	9180	*TRANK1*	8910
FBN1	8616	*UBR5*	8400
FBN2	8739	*UTP20*	8358
FBN3	8556	*VPS13A*	9210
FRY	9177	*VWF*	8442
FRYL	9042	*WDFY3*	9021
GOLGB1	9204	*WDR87*	8739
HRNR	8553	*WNK1*	8502
HTT	9171	*ZAN*	8439
KALRN	8961	*ZFHX3*	8370
KMT2C	8856	*ZZEF1*	8886

Table S2. Model quality validation of otogelin predicted 3D protein structural model.

Protein	Modelled length	Modelling method	Evaluation method				
			Molprobity Score	ERRAT	ProSA-Web	QMEANDisCo	DeepUMQA (lDDT)
OTOG	2925	AlphaFold2	1.29	92.71	-15.56	0.42 ± 0.05	50.26

Table S3. Protein stability change and AlphaMissense prediction caused by missense variants in the otogelin model.

**OTOG variant**	**Stability predictor**			**AlphaMissense**
	**mCSM-stability (kcal/mol)**	**DynaMut2 (kcal/mol)**	**PremPS (kcal/mol)**	
**p.Val141Met**	-0.801 (Destabilizing)	-0.67 (Destabilizing)	1.33 (Destabilizing)	0.7439 (likely pathogenic)
**p.Val269Ile**	-0.391 (Neutral)	-0.71 (Destabilizing)	-0.47 (Neutral)	0.0748 (likely benign)
**p.Pro1240Leu**	-0.449 (Neutral)	-0.49 (Neutral)	0.52 (Destabilizing)	0.3395 (likely benign)
**p.Arg1353Gln**	-0.059 (Neutral)	-0.47 (Neutral)	0.28 (Neutral)	0.0802 (likely benign)
**p.Leu1794Pro**	-0.653 (Destabilizing)	-0.03 (Neutral)	0.41 (Neutral)	0.0659 (likely benign)
**p.His1952Tyr**	0.651 (Stabilizing)	1.52 (Stabilizing)	-0.34 (Neutral)	0.0713 (likely benign)
**p.Ala2037Val**	-0.59 (Destabilizing)	-0.75 (Destabilizing)	-0.39 (Neutral)	0.0939 (likely benign)
**p.Arg2072His**	-0.922 (Destabilizing)	-1.21 (Destabilizing)	0.45 (Neutral)	0.1119 (likely benign)
**p.Arg2556Gln**	0.31 (Neutral)	0.32 (Neutral)	0.53 (Destabilizing)	0.0893 (likely benign)
**p.Gln2621Lys**	-0.12 (Neutral)	0.12 (Neutral)	0.04 (Neutral)	0.1024 (likely benign)
**p.Arg2691Cys**	-0.356 (Neutral)	0.13 (Neutral)	0.46 (Neutral)	0.4357 (ambiguous)
**p.Val2734Met**	-0.242 (Neutral)	-0.47 (Neutral)	0.46 (Neutral)	0.1509 (likely benign)
**p.Arg2802His**	-1.412 (Destabilizing)	-1.18 (Destabilizing)	0.26 (Neutral)	0.0869 (likely benign)

Table S3. The otogelin protein positions have been annotated using the reference sequence NP_001264198.1. For PremPS, $\Delta\Delta G_{pred} < 0.0$ indicates a stabilizing mutation although for DynaMut2 and mCSM, $\Delta\Delta G_{pred} > 0.0$ indicates a stabilizing mutation. Mutations are classified as neutral mutations when $-0.5 < \Delta\Delta G_{pred} < 0.5$.

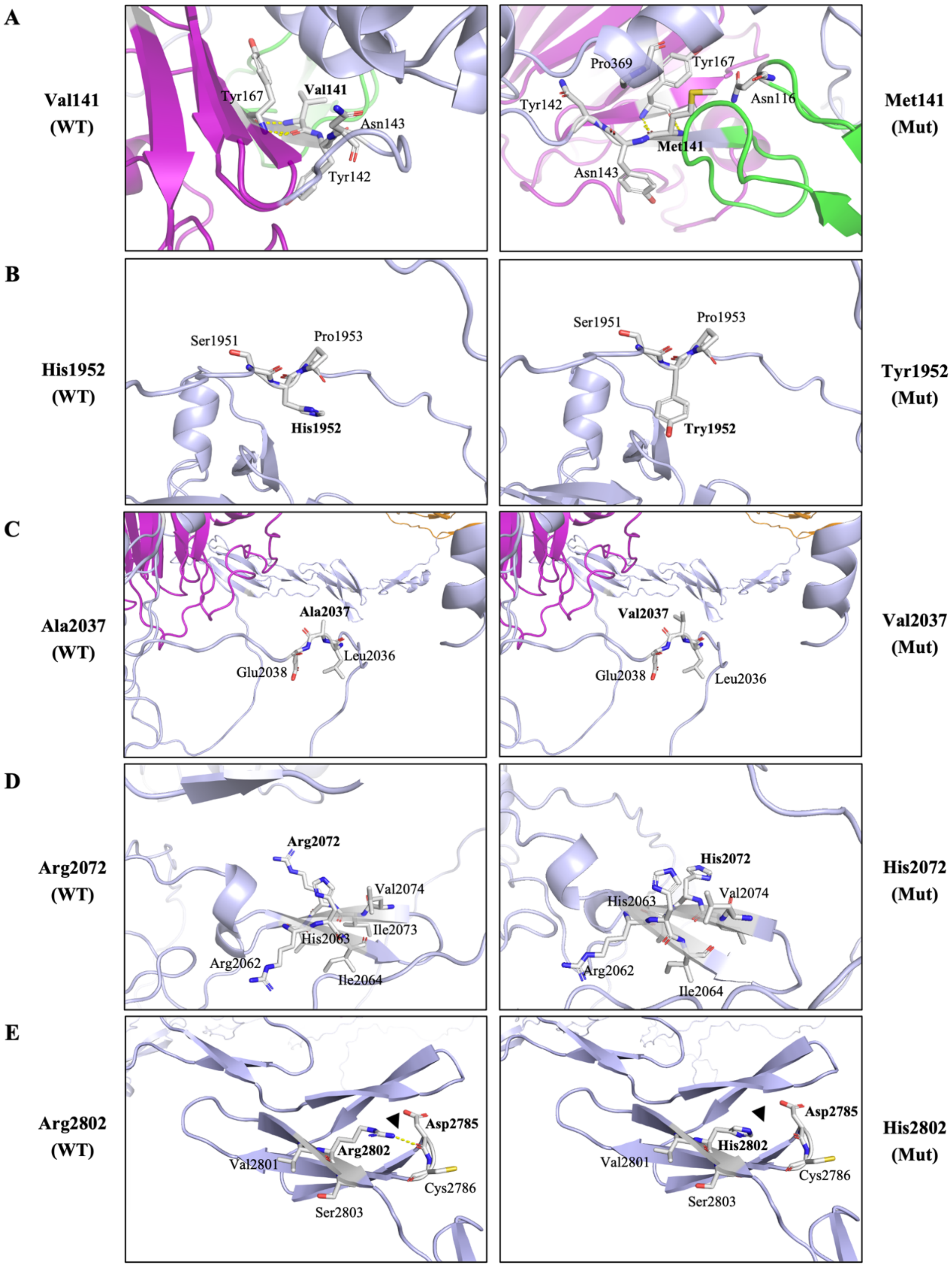

Figure S1. Predicted effect of variants on the otogelin structural model. The 5 variants predicted a change in protein stability (ΔΔG), according mCSM-stability, DynaMut2 and PremPS tools. A) Val141 residue close to EGF-like (Epidermal growth factor-like) domain of wild type (WT) otogelin (left) and NP_001264198.1:p.(Val141Met) mutant (MUT) (right). B) His1952 residue in a WT otogelin disordered interdomain (left) and NP_001264198.1:p.(His1952Tyr) mutant (right). C) Ala2037 residue in a WT otogelin disordered interdomain (left) and NP_001264198.1:p.(Ala2037Val) mutant (right). D) Arg2072 residue in a WT otogelin interdomain (left) and in the NP_001264198.1:p.(Arg2072His) mutant (right). E) Residue

Arg2802 in the interdomain near to the CTCK domain of otogelin WT (left) and NP_001264198.1:p.(Arg2802His) mutant (right). A polar interaction between Arg2802 and Asp2785 is missed. Yellow dashed lines represent polar interactions between residues within 4 Å from the mutated residue. Black triangles show new or missing bonds.
